# Polybrominated Diphenyl Ethers and Cryptorchidism: Confounding or Cause and Effect?

**DOI:** 10.1289/ehp.11052

**Published:** 2008-05

**Authors:** Marek Banasik, Marcia L. Hardy, Todd Stedeford

**Affiliations:** Institute of Public Health and Environmental Protection, Warsaw, Poland, E-mail: iphep.banasik@gmail.com; Health, Safety & Environment, Albemarle Corporation, Baton Rouge, Louisiana

Main et al. (2007) reported that elevated levels of polybrominated diphenyl ethers (PBDEs) in breast milk, but not placenta, were associated with congenital cryptorchidism in Danish and Finnish boys. For the Danish cohort, the researchers used a case–cohort design in which biological samples were collected from all study participants, whereas for the Finnish cohort, they used a nested case–control design. The matching criteria used for Finnish mothers who gave birth to boys with cryptorchidism (measured at birth) and matched Finnish controls included the following: parity, smoking (yes/no), diabetes (yes/no), gestational age (± 7 days), and date of birth (± 14 days). However, Main et al. (2007) did not provide information on adjustments made to account for the type/severity of diabetes (Virtanen et al. 2006), smoking habits (Kurahashi et al. 2005), alcohol consumption (Damgaard et al. 2007), cesarean section (Hjertkvist et al. 1989; Kurahashi et al. 2005), and other factors that may be associated with cryptorchidism.

Interestingly, Main et al. did note that approximately 12% and 18% of breast milk samples and placentas, respectively, were obtained from diabetic Finnish mothers of cryptorchid boys, compared with 0% of controls (Main et al. 2007), but they conducted no further inquiry on this discrepancy. Damgaard et al. (2007) previously evaluated this same Danish–Finnish cohort and adjusted for confounders and effect modifiers for differences observed based on country, smoking, caffeine intake, alcohol consumption (including binge-drinking episodes), social class, maternal age, parity, maturity, and birth weight. Damgaard et al. (2007) noted an association between maternal alcohol consumption (≥ 5 drinks/week) and transient cryptorchidism. Despite these findings, Main et al. (2007) provided no information on stratified alcohol consumption or other potential confounders, such as caffeine intake or social class.

Main et al. (2007) concluded that an association exists between PBDE levels in breast milk and cryptorchidism in boys, but we should be cautious of this interpretation, given that Main et al. (2007) may not have adequately addressed potential confounders.
